# Simulation-Based Education of Health Workers in Low- and Middle-Income Countries: A Systematic Review

**DOI:** 10.9745/GHSP-D-24-00187

**Published:** 2024-12-20

**Authors:** Samuel J.A. Robinson, Angus M.A. Ritchie, Maurizio Pacilli, Debra Nestel, Elizabeth McLeod, Ramesh Mark Nataraja

**Affiliations:** a Department of Paediatrics, Monash University, Melbourne, Victoria, Australia.; b Department of Surgery, Monash University, Melbourne, Victoria, Australia.; c Department of Paediatric Surgery and Monash Children’s Simulation, Monash Children’s Hospital, Melbourne, Victoria, Australia.; d Department of Surgery (Austin Precinct), University of Melbourne, Melbourne, Victoria, Australia.; e Department of Paediatric and Neonatal Surgery, Royal Children’s Hospital, Melbourne, Victoria, Australia.

## Abstract

**Introduction:**

Simulation-based education (SBE) is increasingly used to improve clinician competency and patient care and has been identified as a priority by the World Health Organization for low- and middle-income countries (LMICs). The primary aim of this review was to investigate the global distribution and effectiveness of SBE for health workers in LMICs. The secondary aim was to determine the learning focus, simulation modalities, and additional evaluation conducted in included studies.

**Methods:**

A systematic review was conducted following the Preferred Reporting Items for Systematic Reviews and Meta Analysis guidelines, searching Ovid (Medline, Embase, and Emcare) and the Cochrane Library from January 1, 2002, to March 14, 2022. Primary research studies reporting evaluation at Level 4 of The Kirkpatrick model were included. Studies on simulation-based assessment and validation were excluded. Quality and risk-of-bias assessments were conducted using appropriate tools. Narrative synthesis and descriptive statistics were used to present the results.

**Results:**

A total of 97 studies were included. Of these, 54 were in sub-Saharan Africa (56%). Forty-seven studies focused on neonatology (48%), 29 on obstetrics (30%), and 16 on acute care (16%). Forty-nine used mannequins (51%), 46 used scenario-based simulation (47%), and 21 used synthetic part-task trainers (22%), with some studies using more than 1 modality. Sixty studies focused on educational programs (62%), while 37 used SBE as an adjunct to broader interventions and quality improvement initiatives (38%). Most studies that assessed for statistical significance demonstrated at least partial improvement in Level 4 outcomes (75%, n=81).

**Conclusion:**

SBE has been widely applied to improve outcomes in a variety of contexts across LMICs. Modalities of simulation are typically low-technology versions. However, there is a lack of standardized reporting of educational activities, particularly relating to essential features of SBE. Further research is required to determine which approaches are effective in specific contexts.

## Abstract (precis)

Simulation-based education has been widely applied in low- and middle-income countries and has been shown to improve outcomes in a variety of contexts.

## Teaser

**Key Findings:**

- Simulation-based education (SBE) has been successfully applied to improve outcomes in a variety of contexts across low- and middle-income countries (LMICs), with most studies demonstrating at least a partial improvement in outcomes.
- Simulation modalities are most commonly low-technology modalities, including mannequins and scenario-based simulation.
- Learning methods are often reported insufficiently to judge against reporting standards which have been predominantly developed in high-income countries.

**Key Implications:**

- Further research is needed in LMICs and into specific SBE modalities.
- Improved reporting of SBE is needed, which may require greater representation and consultation of LMICs in the development of a future global consensus on reporting.

## INTRODUCTION

The World Health Organization (WHO) predicts a global shortfall of 10 million health workers by 2030, with low- and middle-income countries (LMICs) most affected.^1^ Underinvestment in training and a mismatch between workforce strategy and health needs are contributing to this shortage. Consequently, numerous strategies have been developed to improve training and retention of health workers. Simulation-based education (SBE) is an approach identified as a priority by the WHO and other stakeholders.^2^^–^^4^

In 2004, Gaba defined simulation as a technique “used to replace or amplify real experiences with guided experiences that evoke or replicate substantial aspects of the real world in a fully interactive manner.”^5^ Increasing awareness of medical errors has catalyzed interest in simulation as an educational method to improve clinician competency and patient safety.^6^ Subsequently, there has been an increase in the published literature focusing on SBE and an expansion of relevant professional bodies.^7^^,^^8^ Numerous studies support the use of SBE to improve knowledge, confidence, and skills.^9^^,^^10^ Additional benefits for low-resource settings include training entry-level health workers and traditional providers.^11^ Importantly, simulation technology and educational efficacy should not be conflated, as research suggests that low-technology simulation can be equally effective as high-technology simulation.^12^^–^^14^

High-income countries continue to be the predominant source of publications and SBE guidelines.^7^^,^^15^^,^^16^ In contrast, little is known about the landscape of SBE across LMICs. It has been suggested that LMIC settings may benefit from SBE, particularly in the context of relatively high rates of adverse events resulting from unsafe experiences in the hospital setting.^17^^,^^18^ Despite this, there is an indication that SBE is underused in LMICs.^18^ A scoping review by Puri et al. identified 203 studies reporting simulation initiatives in LMICs.^18^ Of these, only 85 used simulation in training. The most reported educational modality was low-technology mannequins. There is also limited information available regarding how SBE programs are evaluated in LMICs. A more comprehensive understanding of how SBE has been used in LMICs may serve as useful information for educators and researchers looking to implement this approach.

Little is known about the landscape of SBE across LMICs.

The primary aim of this review was to investigate the global distribution and effectiveness of SBE for health workers in LMICs, specifically those initiatives that evaluate program outcomes. The secondary aim was to determine the learning focus, simulation modalities, and additional evaluation in each included study.

## METHODS

Three authors (SR, AR, and RN) completed this review following the Preferred Reporting Items for Systematic Reviews and Meta Analysis guidelines.^19^ They were additionally informed by the guidelines for Synthesis Without Meta-analysis.^20^ The systematic review was registered on Prospero (CRD42022354079).

### Eligibility Criteria

Primary research studies that trained health workers or health students in LMICs and were published in English between January 1, 2002, and March 14, 2022, were included. Reviewers were guided by Gaba’s definition of simulation and the Healthcare Simulation Dictionary.^5^^,^^21^ The World Bank’s LMIC classification and the WHO health worker classification were also used.^22^^,^^23^ Studies were required to quantitatively evaluate at Level 4 of the Kirkpatrick model.

Studies using simulation exclusively for assessment (without an associated educational initiative) were excluded. These were distinguished from programs that incorporated measurements of student performance as an evaluation of a learning program.^24^ It should be noted that while both assessment and evaluation appear similar, they serve different purposes and have different implications for learners.^24^ Learner assessment using simulation has the potential to create an environment of performance anxiety, which may conflict with the important principle of psychological safety in SBE. It is for these reasons we separated SBE from simulation-based assessment. In practice, the balance between education and assessment needs to be managed carefully. Additional exclusion criteria are listed in Table 1.

**TABLE 1.** Exclusion Criteria for a Systematic Review of Simulation-Based Education in Low- and Middle-Income Countries

General exclusions	Full-text unavailable in English Outside publication date limits Conference abstract Full-text unavailable Review article Educational methods or learning population unclear or poorly defined Program duplicate
Setting exclusions	High-income country Non-health care setting Non-human participants
Intervention exclusions	No educational intervention Educational intervention without live, clinically relevant simulation-based education Simulation without an educational component Validation of simulator outside of a learning program Non-interventional radiology and pathology skills (e.g., interpretation of investigations) Training in basic sciences, including anatomy Training in epidemiology, public health, or policy, including tabletop exercises
Evaluation exclusions	Absence of evaluation or assessment of outcomes or results Unable to demonstrate Kirkpatrick’s Level 4

The Kirkpatrick model is a widely applied evaluation framework and includes 4 levels: reaction, learning, behavior, and results (Table 2).^25^ Evaluation of results (Level 4) has been identified as a priority for SBE research moving forward.^26^ However, it remains difficult to evaluate to this level.^25^^,^^27^^,^^28^ Importantly, successful evaluation at Level 4 is not causally related to other levels, and each level should be considered individually.^29^^,^^30^ The model’s focus on summative evaluation is another concern, which potentially neglects investigations of how changes occur.^29^^,^^31^ However, it has been recommended by the WHO, with return on investment included as a fifth level.^32^^,^^33^

**TABLE 2.** Levels of the Kirkpatrick Model and Applications to Simulation-Based Education^25^

**Kirkpatrick Level And Description**	**Example**
Level 1–Reaction: How participants react to the program	Learner satisfaction with a laparoscopic surgery training program measured by Likert scales
Level 2–Learning: Changes in attitudes, knowledge, and/or skill	An increase in learner’s knowledge regarding laparoscopic surgery following a training program
Level 3–Behavior: Changes in behavior	Increased use of laparoscopic surgery by learners following a training program
Level 4–Results: Changes in final results/outcomes	Reduced length of stay for patients following a laparoscopic surgery training program for learners

For this review, we required that Level 4 evaluations were clearly linked to patient outcomes rather than being inferred from context. As an example, our research laboratory conducted a study using simulation training for air enema in pediatric intussusception.^34^ The length of patient hospital stay was a Level 4 outcome for this study, which was subsequently included. The reduction in the operative rate could also be considered a Level 4 outcome, but this requires additional clinical knowledge regarding the relative advantages of nonoperative approaches. Given it was not feasible for reviewers to have expert knowledge of all the disciplines included in the review, evaluations that changed a treatment approach but did not have a direct link to patient outcomes were excluded.

### Information Sources and Search Strategy

Four databases were searched based on consultation with 2 qualified librarians and used keywords and MeSH terms, including all LMIC names (Supplement 1). The reference management programs used were Endnote and Covidence (Veritas Health Innovation, Melbourne, Australia).

### Selection Process

SR and AR independently screened study titles and abstracts. Conflicts were resolved through discussion and consultation with RN. Except for the deduplication process of Endnote and Covidence, no automation tools were used.

### Data Collection Process

Data relating to the Kirkpatrick model and simulation modalities were extracted independently by SR and AR using Covidence. The remaining data were extracted by SR, with ongoing consultation of AR and RN.

### Data Items

Data extracted included study settings, educational focus, learner populations, simulation modalities, and Kirkpatrick Level 4 outcome measures, as well as which additional Kirkpatrick levels were evaluated. To avoid skewing data, results of multiple studies of identical programs were aggregated. Included simulation modalities were guided by an established simulation dictionary (Table 3).^21^^,^^35^^,^^36^ Missing or unclear data were recorded as such.

**TABLE 3.** List of Simulation Modalities Adapted From the Health Care Simulation Dictionary^21^

**Simulation Modality**	**Description**
Simulated/standardized patients/participants	A person who has been coached to simulate an actual patient/participant
Role-play^a^	Assuming the part of another during simulation
Scenario-based simulation	A detailed description of a simulation exercise
Mannequins	Life-sized human-like simulators
Synthetic part-task trainer	Part-task trainer consisting of synthetic tissue. A part-task trainer describes a device designed to train just the key elements of the procedure or skill being learned
Laparoscopic bench-trainer	A box (or bench) model used to train laparoscopy^35^
Animal-based part-task trainer	Part-task trainer consisting of animal tissue
Cadaveric part-task trainer	Part-task trainer consisting of cadaveric tissue
Extended reality	Virtual, augmented or mixed reality^36^
Virtual patients	A computer program that simulates real-life clinical scenarios in which the learner acts as a health worker
Computer or screen-based simulation	Modelling of real-life processes with inputs and outputs exclusively confined to a computer. Subsets include extended reality and virtual patients
Hybrid simulation	Combining two or more simulation modalities
Telesimulation	Using the internet to link simulators between an instructor and trainee in different locations
Distributed simulation	Transportable, self-contained simulation sets
In-situ simulation	Simulation taking place in the real clinical setting

a This review differentiated simulated/standardized participants and role-play by requiring that simulated/standardized participants be trained/actors.

Outcomes were judged by SR as to whether they achieved statistically significant improvement to the Level 4 outcome measure. Partial improvement was defined as an improvement in some measures, settings, or time points. It should be noted that partial improvement is not necessarily inferior to complete improvement, as studies observing partial improvement may have included more outcome measures. A single standardized metric for outcomes was unsuitable due to study heterogeneity. Missing or unclear data were noted as unspecified.

### Quality Assessment and Risk of Bias

Given the variation in study designs, several quality assessment and risk-of-bias tools were used: the National Institutes of Health before-after (pre-post) quality assessment tool, the Risk of Bias in Non-randomized Studies of Interventions, the Risk of Bias 2 tool for cluster-randomized trials, and the Risk of Bias 2 tool for randomized trials.

### Synthesis Methods

Descriptive statistics and narrative synthesis were used due to study heterogeneity.^37^ Studies were divided into educational programs, which focus exclusively on training, and broader interventions using SBE, which usually included additional quality improvement strategies beyond education. Within these categories, they were further separated into standardized programs, which involve standardized curricula previously used in different settings, and independent programs, which are implemented in a single context or study. For example, Helping Babies Breathe (HBB) is a program initially designed by the American Academy of Pediatrics but has since been applied in many settings, so it was classified as a standardized program.^38^ Conversely, any study using a novel simulator or simulation program would be classified as an independent program.

Other characteristics were tabulated and graphed using GraphPad Prism version 10 (GraphPad Software Inc., MA, USA) and Microsoft Excel version 16 (Microsoft Corporation, WA, USA).

## RESULTS

### Study Selection

**FIGURE 1:**
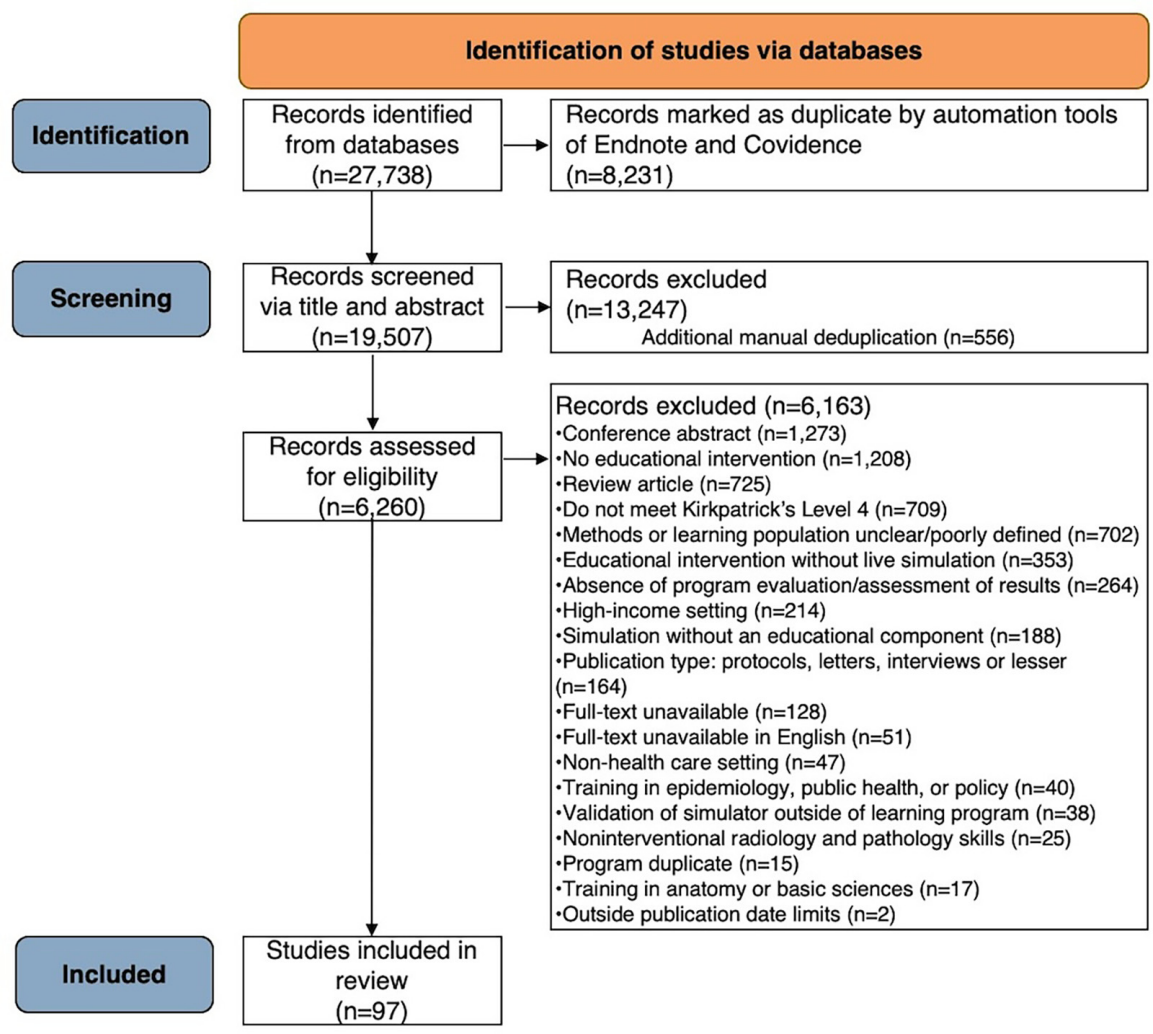
PRISMA Flowchart for a Systematic Review of Simulation-Based Education in Low- and Middle-Income Countries

The initial search yielded 27,738 records. After removing duplicates and screening, 97 studies were included (Figure 1). Included studies are listed in Supplement 2.

### Quality Assessment and Risk of Bias in Studies

Studies assessed using the National Institutes of Health tool included 29 good quality, 35 fair quality, and 12 poor quality articles. Of those assessed using risk-of-bias tools, 8 studies were at low risk of bias and 13 at moderate risk (or “some concerns”).

### Study Characteristics

#### Geographic Distribution

**FIGURE 2:**
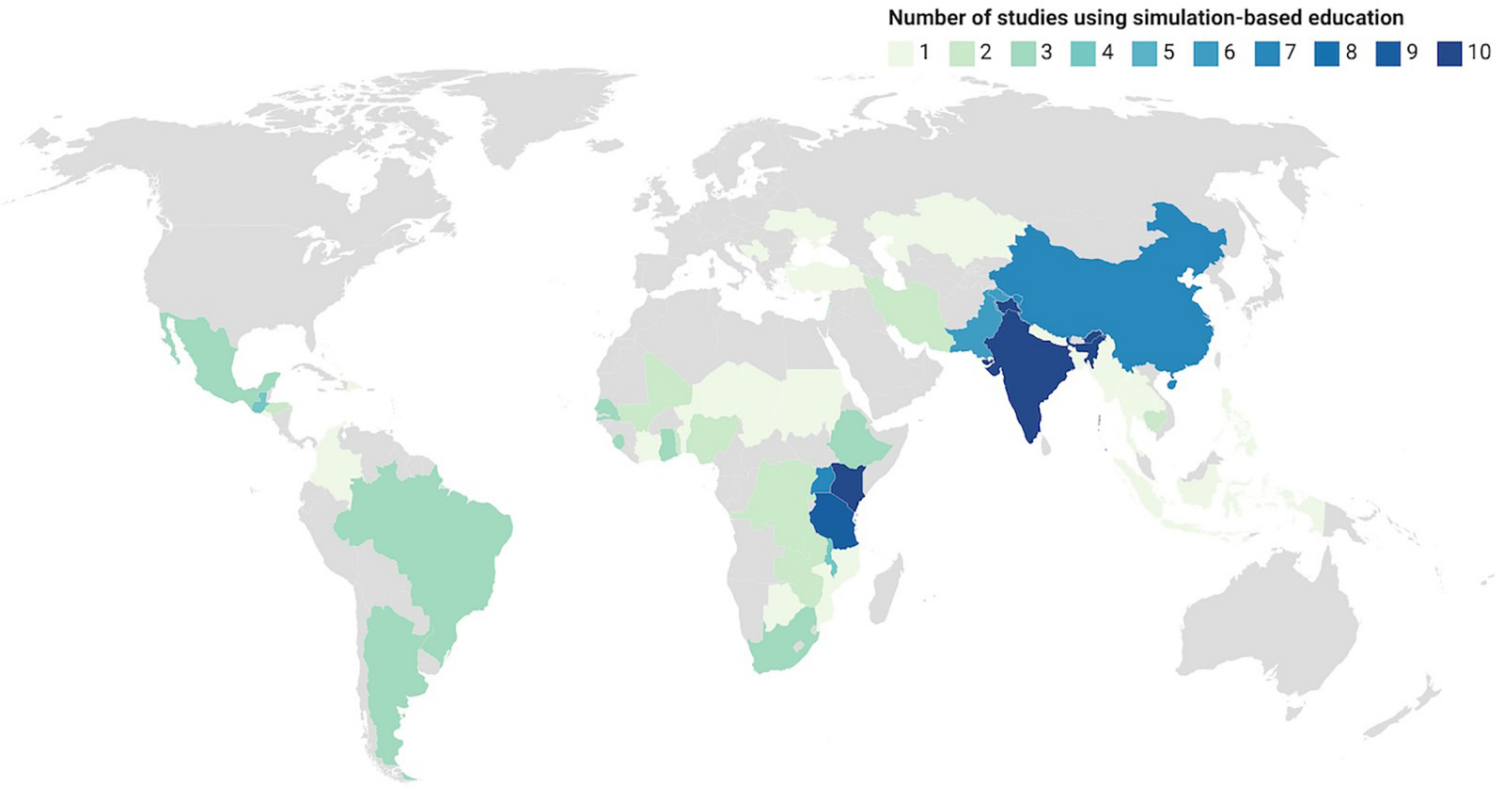
Map of Low- and Middle-Income Countries Where Studies Have Evaluated Results of Simulation-Based Education

Studies across 50 LMICs were included, with 54 representing sub-Saharan Africa (56%) and 15 representing South Asia (15%) (Figure 2). Sixty-seven studies included middle-income countries (69%), 23 included low-income countries (24%), and 7 included a combination (7%).

#### Learning Focus and Populations

The most common educational focus was neonatology (48%) or obstetrics (30%) (Table 4). There were 41 studies training doctors (42%) and 34 training nurses (35%). However, 35 studies also included at least 1 unspecified health worker population (36%).

**TABLE 4.** Learning Focus of Studies in a Systematic Review of Simulation-Based Education in Low- and Middle-Income Countries

	**No. (%)(N=97)**
Neonatology	47 (48)
Obstetrics	29 (30)
Acute/critical care	16 (16)
Communication/leadership/team training	13 (13)
Family planning	9 (9)
Surgery	6 (6)
Infectious diseases	3 (3)
Other	4 (4)

#### Simulation Modalities

SBE modalities are listed in Table 5.

**TABLE 5.** Frequency of Reported Modalities of Simulation-Based Education in Low- and Middle-Income Countries

	**No. (%)** **(N=97)**
Mannequin^a^	49 (51)
Scenario-based simulation	46 (47)
Synthetic part-task trainer	21 (22)
Role-play	20 (21)
Unspecified	20 (21)
Hybrid simulation	8 (8)
In-situ simulation	7 (7)
Animal-based part-task trainer	4 (4)
Simulated/standardized patients/participants	2 (2)
Laparoscopic bench trainer	1 (1)
Cadaveric part-task trainer	1 (1)
Extended reality	1 (1)
Telesimulation	1 (1)

a Mannequins were typically low-technology versions.

#### Kirkpatrick Levels

In addition to Kirkpatrick Level 4, 82 evaluated additional program outcomes (85%). Most commonly, this was Level 3 (behavior), which was evaluated by 80 studies (82%). Nine studies evaluated all levels (9%).

### Simulation-Based Education Programs

Simulation has been used in numerous educational programs across LMICs, with 60 relevant studies identified (62%).

Sixty studies reported on simulation being used in educational programs across LMICs.

#### Standardized

Standardized education programs using simulation were described in 36 studies (37%), with 27 including a neonatology focus and 13 with an obstetric focus (some were focused on both areas). The most frequently reported was Helping Babies Breathe (HBB), which incorporates low-technology mannequins and scenario-based simulation to teach neonatal resuscitation.^39^^,^^40^ HBB has been studied across sub-Saharan Africa, South Asia, and Central America.^40^^–^^45^ The program had success in 8 Tanzanian facilities, resulting in a 42% reduction in neonatal mortality.^41^ However, Arlington et al. demonstrated skill decay 4–6 months after HBB training.^46^ This influenced other HBB initiatives to incorporate maintenance training.^42^ A robust evaluation at Haydom Lutheran Hospital, Tanzania, where HBB training was maintained with frequent refresher sessions, demonstrated ongoing improvements in mortality over 5 years after implementation.^47^

To expand the use of HBB, some programs use “train the trainer” (TTT) approaches.^40^^,^^48^ This is a training cascade where skills and knowledge are transferred to trainees, who then teach others.^49^ Goudar et al. successfully initiated the TTT model for HBB in India, resulting in 599 trained birth attendants.^40^ Ultimately, stillbirth reduced from 3% to 2.3% (*P*=.035).

PRONTO, another standardized simulation course, has minimal didactic content and incorporates hybrid simulation using “PartoPants” simulators to train interprofessional teams on providing optimal neonatal and obstetric care during birth.^50^^–^^52^ Combined with volunteers or simulated patients, this simulates the birth process.^51^^,^^52^ PRONTO has been studied in Mexico, Guatemala, India, Ghana, Kenya, and Uganda.^51^^–^^55^ Walker et al. observed a statistically significant 40% lower neonatal mortality rate in Mexico 8 months after intervention.^52^

Other programs incorporating simulations in LMICs include Practical Obstetric Multi-Professional Training,^56^^,^^57^ Advanced Life Support in Obstetrics,^58^ and Advanced Trauma Life Support.^59^

#### Independent

Twenty-four studies described independent educational programs that included simulation (25%).

Paludo et al. described training Brazilian doctors in laparoscopic partial nephrectomy for renal cancer.^60^ The simulation program incorporated 244 hours of virtual reality laparoscopic simulation into the hospital curriculum over 4 years. Among the 124 surgical patients, those operated on by the simulation-trained group were significantly more likely to achieve the “trifecta” of no complications, negative surgical margins, and minimal decrease in renal function (*P*=.007).^60^

Dean et al. studied cataract surgery across 5 sub-Saharan African countries.^61^ A sample of 50 trainee ophthalmologists received either a 5-day simulation program in addition to standard training or standard training exclusively. The program included surgery on synthetic eyes and described feedback, deliberate practice, and reflective learning. Following completion, clinical outcomes and a validated competency assessment were compared between groups, and posterior capsule rupture rates were significantly lower among the simulation group (7.8% vs. 26.6%, *P*<.001). Competency assessment scores were significantly higher after 3 months and 12 months (*P*<.001). It was not until the 15-month assessment that the difference in scores normalized.

### Simulation-Based Education Components of Other Interventions

SBE has been successfully used to complement other quality-improvement strategies.^53^^,^^62^^,^^63^ Thirty-seven (38%) studies were included in this category.

#### Interventions Including Standardized Simulation-Based Training

Seventeen initiatives used SBE as a core component of their intervention (18%). The Emergency Triage Assessment and Treatment Course combined role-play with modular learning of guidelines. Variations of this course have been applied in several settings.^64^^–^^66^ For example, Hands et al. implemented a 5-day program in Sierra Leone training nurses. Subsequently, pediatric mortality and treatment practices improved; however, statistical analysis was not conducted.^64^

Seventeen initiatives used SBE as a core component of their intervention.

Other studies used previously described standardized programs but packaged them alongside other initiatives. For example, Rule et al. studied HBB in Kenya but included policy revision and administrative changes relating to data collection.^43^ After completion of the quality-improvement program, including training 96 providers in HBB, the rate of asphyxia-related mortality decreased by 53% (*P*=.01).

A similar adaptation of PRONTO was reported by Walker et al. in Kenya and Uganda.^53^ The intervention package additionally included data-strengthening strategies, quality improvement collaboratives, and a childbirth checklist. The study was a cluster-randomized trial, where facilities were pair-matched and assigned intervention or control. The findings demonstrated reduced neonatal mortality and stillbirth among the intervention facilities (15.3% vs. 23.3%, *P*<.0001).

Interventions, including social media campaigns and workforce changes, have also been used alongside formalized simulation programs.^54^

#### Interventions Including Independent Simulation-Based Training

Broader interventions, including nonstandardized, independent SBE, were described in 20 studies (21%).

One example by Gill et al. trained traditional birth attendants in Zambia.^67^ The program included teaching a resuscitation protocol using infant mannequins. The second major component of the intervention was the provision of antibiotics and facilitated referral for possible sepsis. Traditional birth attendants received delivery kits for each birth, including resuscitation equipment, medications, and general materials. The program ultimately resulted in a significant reduction in neonatal mortality.

Another program in Kazakhstan involved a variety of interventions, including patient registries and clinician training to encourage patient self-management of chronic diseases.^63^ Role-playing was used to teach and encourage clinicians to engage in supportive dialogue with patients. Among other results, the program successfully improved the management of hypertension, with significant changes in the blood pressure of patients following the program. Specifically, the percentage of patients with blood pressures below 140/90mmHg increased from 24% to 56% (*P*<.001).

### Outcome Improvement

Eighty-one studies tested for statistical significance (84%). Of this subset, 42 (52%) demonstrated a partial improvement in outcomes and 19 (23%) demonstrated a complete improvement. Standardized programs were more likely to be associated with improvement in outcomes when compared to independent programs (78% vs. 71%). Seven studies provided information regarding cost of programs (7%) but did not report comparable measures.

## DISCUSSION

This systematic review found that SBE has been successfully incorporated through standardized and independent programs in LMICs. Studies were most frequently in the fields of neonatology and obstetrics and in sub-Saharan Africa. We believe this is the first study to provide systematic and comprehensive insight into SBE use in LMICs. The findings support the potential for SBE to effectively train health workers in LMICs. Many studies demonstrated significant improvements in patient outcomes following programs.^40^^,^^41^^,^^43^^,^^47^^,^^52^^,^^53^^,^^60^^,^^61^^,^^63^^,^^64^^,^^67^ Innovative approaches, such as TTT, may be effective methods to further improve simulation capacity. The results of this review support using SBE to complement traditional educational approaches in LMICs.

The findings of this review support the potential for SBE to effectively train health workers in LMICs.

The significant representation of neonatology and obstetrics in this review may be due to their acute and interdisciplinary nature, which is particularly suited to SBE.^68^ Funding is likely relevant, with child and maternal health being major recipients of international financing.^69^ This finding differs from the review by Puri et al., where infectious diseases and reproductive health were the most common fields. This difference is likely explained by the inclusion of simulation-based assessment, which may be more suited to these topics.^18^ The significant representation of sub-Saharan Africa in our study is unsurprising given the region’s comparably greater number of LMICs, though this differs again from the findings of the Puri study.^22^

The most frequently reported modalities were mannequins, scenario-based simulation, and synthetic part-task trainers, likely due to their affordability and accessibility as lower-technology approaches. However, low-technology differs from low-efficacy, and low-technology approaches have been identified as a priority in resource-limited settings.^12^^,^^18^ The widespread application of lower-technology modalities suggests the educational value of low-cost simulation is being realized. Puri et al. reported simulated patients as the most frequent modality in LMICs with a significant number of studies involving them as a method of quality assessment. When looking exclusively at SBE, they found mannequins to be most prevalent, which is consistent with our findings.

The number of studies identified (n=97) demonstrates evaluation at Level 4 of the Kirkpatrick model has been achieved in many LMIC settings. However, only 9 studies evaluate all 4 levels, which is consistent with previous reviews of health care education.^28^^,^^70^ Level 3 of the Kirkpatrick model, evaluation of behavior, was also evaluated in the majority of studies.

When discussing SBE broadly, it is difficult to draw definitive conclusions about its efficacy in LMICs based on this review. This is due to the variety of contexts, simulation modalities, and outcome measures included. Consequently, many studies were judged as “partially” effective, an issue with systematic reviews that has long been recognized.^71^ Overlaid upon this is the issue of “causal attribution,” where it is difficult to determine definitively that the program was responsible for outcomes.^25^ Despite this, we found good evidence to suggest SBE can be used in LMICs, with statistically significant improvements evident after numerous initiatives.

Evidence demonstrating the efficacy of standardized programs can be more robust than independent programs as they have been studied in more contexts. In particular, the HBB program has been associated with significant reductions in stillbirth and neonatal mortality. The program also demonstrated the importance of skill maintenance for program efficacy, a finding consistent with previous studies.^18^^,^^46^^,^^47^^,^^72^

Independent programs have proved that effective SBE is also possible outside of standardized structures. Such programs have confirmed the value of integrating training into formalized institutional structures or curricula.^60^ This is consistent with established simulation literature, which highlights curriculum integration as critical.^73^ Despite the benefits of standardized courses, they may not be contextualized appropriately unless necessary modifications are adapted.^74^ Consideration of the educational context is vital to the success of SBE.^73^

This review highlights that SBE is not limited to educational programs and is also used to introduce other initiatives, such as guidelines and protocols, or as an adjunct to additional interventions.^64^

Despite successful applications, it remains difficult to judge what makes an SBE program effective in LMICs. This can be partially attributed to deficits in SBE reporting. Few studies report methods in sufficient detail to facilitate comparison to reporting guidelines, which is unsurprising given both the recency of guideline development and their lack of emphasis on perspectives from LMICs.^15^^,^^16^ Nonetheless, there is typically minimal reporting of simulation design, prebriefing, and debriefing, and limited discussion of educational factors, such as deliberate practice and mastery learning. Issues, including skill maintenance, feedback, and curriculum integration, are discussed sporadically but not to the extent where meaningful comparisons can be made. This is an issue for educators and researchers, given the importance of these factors for educational effectiveness. Ongoing simulation training is necessary to maintain skills. It has been suggested that reflective debriefing is the most important feature of SBE to facilitate learning.^73^ While evidence suggests debriefing may occur differently across different societies and cultures,^75^ it remains important to understand how feedback is given to learners in any context. We also were unable to extract meaningful data regarding other relevant features, including learner groups, facilitator training, and TTT approaches, despite their importance.^73^ We recommend authors improve reporting of SBE initiatives to provide greater detail regarding their approach, which will improve transparency and replicability. This should include the above features of programs, as well as others such as program cost and duration. Such reporting would provide greater insights regarding the features of successful programs. In addition, it may help address misconceptions around SBE, including the idea that it is prohibitively expensive for low-resource settings. We also recommend greater involvement of LMIC perspectives in the development of future guidelines to ensure they are appropriate and consider all settings.

### Strengths and Limitations

The strengths of the review include its comprehensive search strategy, the number of articles screened, and the focus and detail of data extraction. However, the topic required interpretation of definitions that are often contested in the simulation community, such as simulation modalities. For example, simulation scenarios can be described to varying degrees, so determining when studies qualify as scenario-based simulation was difficult. Similarly, determining when a program is describing team training was difficult, so we relied upon this being explicitly articulated by the authors, and equivalent issues were encountered when ascertaining evaluation levels. For example, when training communication skills for doctors providing contraception, determining what classifies as a Level 4 evaluation is debatable. It may be patient satisfaction, contraceptive uptake, pregnancy rates, or pregnancy-related complications. Judging the modalities used in standardized courses was also difficult as some materials are not publicly available. In these situations, modalities were determined based on organizational websites, course resources, and published literature. To minimize any subjectivity, we relied upon the independent consensus of 2 investigators for study inclusion, judgment of Kirkpatrick levels, and simulation modalities, with a third investigator resolving conflicts. The variability of program details reported, as previously discussed, also limited the conclusions that could be drawn.

## CONCLUSION

Ultimately, SBE has been used successfully to train health workers in LMICs across numerous settings. However, there is a need for further research in low-income countries, and the evidence supporting some modalities is greater than others. Reporting varies significantly, with often insufficient information provided to judge against reporting standards. Furthermore, standards are based heavily on research from high-income countries and are rarely referenced in LMICs. This suggests a need to develop and adopt region-specific reporting standards or to include LMICs in the development of a future global consensus. This would ensure standards are appropriate for all settings and would potentially improve their use in LMICs.

## Supplementary Material

GHSP-D-24-00187-Supplement.pdf

## Notes

**Peer Reviewed**

**First Published Online:** November 7, 2024.
