# Supplementary material for: Simulation-Based Education of Health Workers in Low- and Middle-Income Countries: A Systematic Review
Source: Glob Health Sci Pract. 2024 Dec 20;12(6):e2400187. doi: 10.9745/GHSP-D-24-00187 (PMC11666082; doi:10.9745/GHSP-D-24-00187)
Supplement: GHSP-D-24-00187-Supplement.pdf [file GHSP-D-24-00187-Supplement.pdf]

Supplement to: Robinson SJA, Ritchie AMA, Pacilli M, Nestel D, McLeod E, Nataraja RM. Simulation-based education of health workers in low- and middle-income countries: a systematic review. *Glob Health Sci Pract*. 2024;12(6):e2400187. https://doi.org/10.9745/GHSP-D-24-000187

SUPPLEMENT 1. Ovid Medline search strategy for simulation-based education in low- and middle-income countries

#	Search string	# of results
1	exp Simulation Training/ or exp Educational Technology/ or Role Playing/	124710
2	(((Simulat* or scenario*) adj5 (educat* or learn* or teach* or train* or practice or practise or instruct* or course or workshop)) or (SBE or SBME or simulation based)).mp.	27027
3	(roleplay or role play or manikin or mannikin or mannequin or part task trainer or partial task trainer or bench trainer or laparoscop* trainer or bench model or laparoscop* model).mp.	5828
4	Virtual reality/ or Augmented reality/ or Computer simulation/ or User computer interface/ or Clinical competence/ or Laparoscopy/ or Education, Distance/	436177
5	(virtual reality or augment* reality or interactive learning or learning interactive or acting or distance education or virtual education or remote education).mp.	190461
6	exp Simulation Training/ or (educat* or learn* or teach* or train* or practice or practise or instruct* or course or workshop or simulat*).mp.	3570147
7	(4 or 5) and 6	334290
8	Exp Emergency Treatment/ or exp Emergencies/	168376
9	6 and 8	33709
10	((resuscit* or emergency or trauma* or CPR or ALS or APLS or life support) adj5 (educat* or learn* or teach* or train* or practice or practise or instruct* or course or workshop or simulat*)).mp.	23958
11	1 or 2 or 3 or 7 or 9 or 10	497291
12	Developing Countries/ or Global Health/ or Health Equity/	128863
13	(Deprived Countries or Deprived Population or Deprived Populations or Developing Countries or Developing Country or Developing Economies or Developing Economy or Developing Nation or Developing Nations or Developing Population or Developing Populations or Developing World or LAMI Countries or LAMI Country or Less Developed Countries or Less Developed Country or Less Developed Economies or Less Developed Nation or Less Developed Nations or Less Developed World or Lesser Developed Countries or Lesser Developed Nations or LMIC or LMICS or Low GDP or Low GNP or Low Gross Domestic or Low Gross National or Low Income Countries or Low Income Country or Low Income Economies or Low Income Economy or Low Income Nations or Low Income Population or Low Income Populations or Lower GDP or lower gross domestic or Lower Income Countries or Lower Income Country or Lower Income Nations or Lower Income Population or Lower Income Populations or Middle Income Countries or Middle Income Country or Middle Income Economies or Middle Income Nation or Middle Income Nations or Middle Income Population or Middle Income Populations or Poor Countries or Poor Country or Poor Economies or Poor Economy or Poor Nation or Poor Nations or Poor Population or Poor Populations or poor world or Poorer Countries or Poorer Economies or Poorer Economy or Poorer Nations or Poorer Population or Poorer Populations or Third World or Transitional Countries or Transitional Country or Transitional Economies or Transitional Economy or Under Developed Countries or Under Developed Country or under developed nations or Under Developed World or Under Served Population or Under Served	129359

Supplement to: Robinson SJA, Ritchie AMA, Pacilli M, Nestel D, McLeod E, Nataraja RM. Simulation-based education of health workers in low- and middle-income countries: a systematic review. *Glob Health Sci Pract*. 2024;12(6):e2400187. https://doi.org/10.9745/GHSP-D-24-000187

	Populations or Underdeveloped Countries or Underdeveloped Country or underdeveloped economies or underdeveloped nations or underdeveloped population or Underdeveloped World or Underserved Countries or Underserved Nations or Underserved Population or Underserved Populations).ti,ab,kw.	
14	((Resource limited or Low resource) and (Countr* or Nation* or Setting* or Context* or Population* or Econom*)).ti,ab,kw.	13018
15	(Afghanistan or Albania or Algeria or American Samoa or Angola or Argentina or Armenia or Azerbaijan or Bangladesh or Belarus or Belize or Benin or Bhutan or Bolivia or (Bosnia and Herzegovina) or Botswana or Brazil or Bulgaria or Burkina Faso or Burundi or Cabo Verde or Cambodia or Cameroon or Central African Republic or Chad or China or Colombia or Comoros or Congo Dem Rep or Congo Rep or Costa Rica or C*te d*Ivoire or Cuba or Djibouti or Dominica or Dominican Republic or Ecuador or Egypt or Arab Republic of Egypt or Egypt Arab Rep or El Salvador or Equatorial Guinea or Eritrea or Eswatini or Ethiopia or Fiji or Gabon or Gambia or The Gambia or Republic of The Gambia or Georgia or Ghana or Grenada or Guatemala or Guinea or Guinea-Bissau or Guyana or Haiti or Honduras or India or Indonesia or Iran or Islamic Republic of Iran or Iraq or Jamaica or Jamaica or Jordan or Kazakhstan or Kenya or Kiribati or North Korea or Korea Dem People*s Rep or Kosovo or Kyrgyzstan or Kyrgyz Republic or Laos or Lao PDR or Lao People*s Democratic Republic Lebanon or Lesotho or Liberia or Libya or Madagascar or Malawi or Malaysia or Maldives or Mali or Marshall Islands or Mauritania or Mauritius or Mexico or Micronesia Fed Sts or Moldova or Mongolia or Montenegro or Morocco or Mozambique or Myanmar or Namibia or Nepal or Nicaragua or Niger or Nigeria or North Macedonia or Pakistan or Panama or Papua New Guinea or Paraguay or Peru or Philippines or Romania or Russia or Russian Federation or Rwanda or Samoa or (S*o Tom* and Principe) or Senegal or Serbia or Sierra Leone or Solomon Islands or Somalia or South Africa or South Sudan or Sri Lanka or St Lucia or (St Vincent and the Grenadines) or Sudan or Suriname or Syria or Syrian Arab Republic or Tajikistan or Tanzania or Thailand or Timor Leste or Togo or Tonga or Tunisia or Turkey or Turkmenistan or Tuvalu or Uganda or Ukraine or Uzbekistan or Vanuatu or Venezuela or Venezuela RB or Vietnam or (West Bank and Gaza) or Yemen or Republic of Yemen or Zambia or Zimbabwe).ti,ab,kw.	1008512
16	12 or 13 or 14 or 15	1143003
17	(health* or medical or surg*).mp. or exp Health Personnel/ or exp Health occupations/ or exp Students, Health occupations/ or exp Education, Professional/ or exp Health Care Sector/	8547884
18	11 and 16 and 17	11308
19	limit 18 to (english language and yr="2002 -Current")	9559

Supplement to: Robinson SJA, Ritchie AMA, Pacilli M, Nestel D, McLeod E, Nataraja RM. Simulation-based education of health workers in low- and middle-income countries: a systematic review. *Glob Health Sci Pract*. 2024;12(6):e2400187. https://doi.org/10.9745/GHSP-D-24-000187

SUPPLEMENT 2. List of included studies in a systematic review of simulation-based education programs in low- and middle-income countries evaluating at Level 4 of the Kirkpatrick model

Note: References are displayed in the alphabetical order of author names. The numbering of references matches that of the main text.

Author	Year	Country/Setting	Use of simulation-based education	Program Type	Learning Population/s	Simulation Modalities	Educational Focus	Statistically Significant Improvement in Results	Kirkpatrick Levels Evaluated (in Addition to Level 4)	Risk of bias / Quality Assessment Outcome
Afulani[51]	2019	Ghana	Educational program	Standardized	Midwives, Doctors, Nurses	Role-play, Scenario-based, Mannequin, Synthetic PTT, Hybrid simulation	Neonatology; Obstetrics; Communication/ leadership/ team training	Yes	None	Good
Arabi[76]	2018	Sudan	Educational program	Standardized	Midwives	Scenario-based, Mannequin	Neonatology; Obstetrics	Yes	Level 3	Good
Arreola-Risa[77]	2004	Mexico	Educational program	Standardized	Unspecified health workers	Mannequin, Animal PTT	Acute/critical care	Partially	Level 3	Fair
Aziz[78]	2020	China	Educational program	Standardized	Doctors, Nurses	Scenario-based	Neonatology	No	Level 3	Fair
Baumgartner[79]	2021	Uganda	Educational program	Independent	Unspecified health workers	Mannequin	Neonatology; Obstetrics	NA	Level 3	Fair
Bellad[48]	2016	1. India<br>2. Kenya	Educational program	Standardized	Unspecified health workers	Scenario-based, Mannequin	Neonatology	Partially	None	Good
Bender[80]	2016	Kosovo	Educational program	Standardized	Doctors, Nurses	Scenario-based	Neonatology	Partially	None	Fair
Brathwaite[81]	2020	Ghana	Educational program	Standardized	Doctors, Nurses, Midwives	Role-play, Scenario-based, Mannequin	Neonatology	Partially	Level 2; Level 3	Fair
Carlo[82]	2010	1. Argentina<br>2. Democratic Republic of Congo<br>3. Guatemala<br>4. India<br>5. Pakistan<br>6. Zambia	Educational program	Standardized	Traditional Birth Attendants, Nurses, Midwives, Doctors	Role-play, Scenario-based	Neonatology	Partially	Level 2; Level 3	Poor
Carvalho[83]	2019	Brazil	Educational program	Independent	Doctors, Nurses	Scenario-based, Mannequin	Other: Neurology	No	Level 3	Good
Chan[63]	2020	Kazakhstan	Broader intervention/initiative	Independent	Unspecified health workers	Role-play	Other: Cardiology	Partially	Level 3	Good

Supplement to: Robinson SJA, Ritchie AMA, Pacilli M, Nestel D, McLeod E, Nataraja RM. Simulation-based education of health workers in low- and middle-income countries: a systematic review. *Glob Health Sci Pract*. 2024;12(6):e2400187. https://doi.org/10.9745/GHSP-D-24-000187

Author	Year	Country/Setting	Use of simulation-based education	Program Type	Learning Population/s	Simulation Modalities	Educational Focus	Statistically Significant Improvement in Results	Kirkpatrick Levels Evaluated (in Addition to Level 4)	Risk of bias / Quality Assessment Outcome
Chang[84]	2019	Malawi	Broader intervention/initiative	Standardized	Doctors, Nurses, Midwives	Scenario-based	Neonatology; Obstetrics; Communication/ leadership/ team training	Partially	Level 3	Good
Charafeddine[85]	2016	Lebanon	Educational program	Standardized	Doctors, Nurses, Midwives, Allied health	Scenario-based, Mannequin	Neonatology	Partially	Level 1; Level 2	Fair
Charyeva[86]	2015	Nigeria	Educational program	Independent	Other	Synthetic PTT	Family planning	Partially	Level 3	Fair
Cioe-Pena[87]	2017	El Salvador	Broader intervention/initiative	Standardized	Doctors, Nurses	Unspecified simulation modality	Acute/critical care	No	Level 2; Level 3	Fair
Clark[88]	2012	Sierra Leone	Broader intervention/initiative	Standardized	Nurses	Role-play, Scenario-based	Acute/critical care	Yes	None	Fair
Crofts[57]	2015	Zimbabwe	Educational program	Standardized	Doctors, Nurses, Midwives, Allied health	Scenario-based, Mannequin, Synthetic PTT	Obstetrics	NA	Level 3	Good
Cummings[89]	2017	Uganda	Broader intervention/initiative	Independent	Unspecified health workers	Unspecified simulation modality	Other: Diagnosis of severe illness	No	Level 3	Fair
Das[90]	2022	Bangladesh	Educational program	Standardized	Nurses, Midwives, Allied health	Scenario-based, Mannequin, In-situ simulation	Neonatology; Communication/ leadership/ team training	NA	None	Poor
Das[91]	2019	India	Broader intervention/initiative	Standardized	Doctors, Nurses, Nurse/midwives	Mannequin	Neonatology; Obstetrics	Partially	Level 1; Level 2; Level 3	Good
Dean[61]	2021	1. Kenya<br>2. Tanzania<br>3. Uganda<br>4. Zimbabwe<br>3. Uganda<br>4. Zimbabwe	Educational program	Independent	Doctors	Synthetic PTT	Surgery (Ophthalmology)	Yes	Level 2; Level 3	Low

Supplement to: Robinson SJA, Ritchie AMA, Pacilli M, Nestel D, McLeod E, Nataraja RM. Simulation-based education of health workers in low- and middle-income countries: a systematic review. *Glob Health Sci Pract*. 2024;12(6):e2400187. https://doi.org/10.9745/GHSP-D-24-000187

**Author**	**Year**	**Country/Setting**	**Use of simulation-based education**	**Program Type**	**Learning Population/s**	**Simulation Modalities**	**Educational Focus**	**Statistically Significant Improvement in Results**	**Kirkpatrick Levels Evaluated (in Addition to Level 4)**	**Risk of bias / Quality Assessment Outcome**
De Melo[92]	2021	Brazil	Educational program	Independent	Doctors	Simulated/standardized patients/participants, Scenario-based, Synthetic PTT, Hybrid simulation, In-situ simulation	Obstetrics	No	Level 3	Fair
Dresang[58]	2015	1. Colombia<br>2. Guatemala<br>3. Honduras<br>4. Tanzania	Educational program	Standardized	Doctors, Nurses, Nurse/midwives, Other	Scenario-based, Mannequin	Obstetrics	Partially	Level 2; Level 3	Fair
Dumont[93]	2013	1. Senegal<br>2. Mali	Broader intervention/initiative	Standardized	Doctors, Midwives	Scenario-based	Obstetrics	Partially	Level 3	Some concerns
Egenberg[56]	2017	Tanzania	Educational program	Standardized	Nurses, Midwives, Doctors, Other	Role-play, Scenario-based, Mannequin, Synthetic PTT, Hybrid simulation	Neonatology; Obstetrics	Partially	Level 3	Good
Ellard[94]	2016	Tanzania	Educational program	Independent	Clinical Officers, Nurse/midwives	Unspecified simulation modality	Neonatology; Obstetrics; Communication/ leadership/ team training	No	Level 1; Level 2; Level 3	Fair
Evans[95]	2018	Uganda	Educational program	Standardized	Unspecified health workers	Scenario-based, Mannequin	Neonatology; Obstetrics; Communication/ leadership/ team training	Partially	Level 2; Level 3	Some concerns
Fikree[96]	2020	Ethiopia	Broader intervention/initiative	Independent	Unspecified health workers	Synthetic PTT	Family planning	Partially	Level 3	Poor

Supplement to: Robinson SJA, Ritchie AMA, Pacilli M, Nestel D, McLeod E, Nataraja RM. Simulation-based education of health workers in low- and middle-income countries: a systematic review. *Glob Health Sci Pract*. 2024;12(6):e2400187. https://doi.org/10.9745/GHSP-D-24-000187

Author	Year	Country/Setting	Use of simulation-based education	Program Type	Learning Population/s	Simulation Modalities	Educational Focus	Statistically Significant Improvement in Results	Kirkpatrick Levels Evaluated (in Addition to Level 4)	Risk of bias / Quality Assessment Outcome
Gandhi Bhatt[97]	2021	India	Educational program	Independent	Doctors	Unspecified simulation modality	Surgery (Ophthalmology)	Yes	Level 3	Good
Geldenhuys[98]	2020	South Africa	Broader intervention/initiative	Independent	Doctors	Unspecified simulation modality	Acute/critical care	Partially	Level 3	Poor
Gill[67]	2011	Zambia	Broader intervention/initiative	Independent	Traditional Birth Attendants	Mannequin	Neonatology	Partially	Level 3	Some concerns
Goudar[40]	2013	India	Broader intervention/initiative	Standardized	Unspecified health workers	Scenario-based, Mannequin	Neonatology	Partially	Level 3	Good
Goudar[99]	2012	India	Broader intervention/initiative	Standardized	Other, Nurse/midwives, Doctors	Scenario-based, Mannequin	Neonatology	Partially	Level 2; Level 3	Some concerns
Gueye[100]	2016	Senegal	Broader intervention/initiative	Independent	Nurses, Midwives, Unspecified health workers	Synthetic PTT	Family planning	NA	Level 3	Good
Hands[64]	2020	Sierra Leone	Broader intervention/initiative	Standardized	Doctors, Nurses	Role-play	Acute/critical care	NA	Level 3	Fair
Hashmi[101]	2013	Pakistan	Broader intervention/initiative	Independent	Doctors	Unspecified simulation modality	Acute/critical care	Partially	None	Fair
Hawkins[102]	2021	Botswana	Broader intervention/initiative	Independent	Doctors, Nurses	Role-play	Family planning	NA	Level 1; Level 3	Poor
Houy[103]	2017	Cambodia	Educational program	Independent	Traditional Birth Attendants, Other, Midwives, Other	Mannequin	Neonatology; Obstetrics	Partially	None	Fair
Hu[104]	2016	China	Broader intervention/initiative	Independent	Unspecified health workers	In-situ simulation, Unspecified simulation modality	Obstetrics	Yes	Level 3	Fair
Innerdal[105]	2020	Mali	Educational program	Standardized	Unspecified health workers	Scenario-based, Mannequin	Neonatology	Partially	Level 3	Good
Irimu[66]	2012	Kenya	Broader intervention/initiative	Standardized	Unspecified health workers, Clinical Officers, Nurses, Doctors	Role-play, Scenario-based, Mannequin	Acute/critical care	NA	Level 3	Fair

Supplement to: Robinson SJA, Ritchie AMA, Pacilli M, Nestel D, McLeod E, Nataraja RM. Simulation-based education of health workers in low- and middle-income countries: a systematic review. *Glob Health Sci Pract*. 2024;12(6):e2400187. https://doi.org/10.9745/GHSP-D-24-000187

Author	Year	Country/Setting	Use of simulation-based education	Program Type	Learning Population/s	Simulation Modalities	Educational Focus	Statistically Significant Improvement in Results	Kirkpatrick Levels Evaluated (in Addition to Level 4)	Risk of bias / Quality Assessment Outcome
Jones-Konneh[106]	2017	Sierra Leone	Educational program	Independent	Unspecified health workers	Simulated/standardized patients/participants, Scenario-based	Infectious diseases	NA	None	Poor
Jorro-Baron[107]	2021	Argentina	Broader intervention/initiative	Independent	Unspecified health workers	Role-play	Communication/ leadership/ team training	No	Level 2; Level 3	Low
Joudeh[55]	2021	India	Educational program	Standardized	Nurse/midwives	Scenario-based, Mannequin, Synthetic PTT, Hybrid simulation, Unspecified simulation modality	Neonatology; Obstetrics; Communication/ leadership/ team training	Partially	Level 3	Fair
Kamath-Rayne[44]	2017	Honduras	Educational program	Standardized	Doctors, Nurses	Scenario-based, Mannequin	Neonatology	No	Level 3	Good
Kestler[108]	2020	Guatemala	Broader intervention/initiative	Standardized	Unspecified health workers	Scenario-based, Mannequin, Synthetic PTT, Hybrid simulation, Unspecified simulation modality	Neonatology; Obstetrics; Communication/ leadership/ team training	No	Level 3	Low
Matendo[109]	2011	Democratic Republic of Congo	Broader intervention/initiative	Standardized	Traditional Birth Attendants, Nurses, Midwives	Mannequin	Neonatology	Yes	None	Low
Michelangelo[110]	2020	Argentina	Educational program	Independent	Nurse/midwives, Allied health	Role-play, Scenario-based, Unspecified simulation modality	Infectious diseases	Yes	Level 3	Good
Miller[111]	2012	Pakistan	Educational program	Independent	Traditional Birth Attendants	Role-play, Mannequin	Neonatology; Obstetrics	Yes	Level 2; Level 3	Good

Supplement to: Robinson SJA, Ritchie AMA, Pacilli M, Nestel D, McLeod E, Nataraja RM. Simulation-based education of health workers in low- and middle-income countries: a systematic review. *Glob Health Sci Pract*. 2024;12(6):e2400187. https://doi.org/10.9745/GHSP-D-24-000187

Author	Year	Country/Setting	Use of simulation-based education	Program Type	Learning Population/s	Simulation Modalities	Educational Focus	Statistically Significant Improvement in Results	Kirkpatrick Levels Evaluated (in Addition to Level 4)	Risk of bias / Quality Assessment Outcome
Mogilevkina[112]	2022	Ukraine	Educational program	Standardized	Doctors, Midwives	Mannequin	Obstetrics	Partially	Level 3	Moderate
Molyneux[65]	2006	Malawi	Broader intervention/initiative	Standardized	Unspecified health workers	Role-play	Acute/critical care	NA	Level 3	Fair
Mould-Millman[113]	2019	South Africa	Educational program	Independent	Other	In-situ simulation, Unspecified simulation modality	Acute/critical care	NA	Level 1; Level 2; Level 3	Fair
Mubeen[114]	2021	Pakistan	Educational program	Standardized	Midwives	Scenario-based, Mannequin	Neonatology	No	Level 3	Good
Mugore[115]	2016	Togo	Broader intervention/initiative	Independent	Nurses, Midwives	Synthetic PTT	Family planning	NA	Level 3	Poor
Mukhtar-Yola[116]	2018	Nigeria	Broader intervention/initiative	Standardized	Doctors, Midwives	Scenario-based, Mannequin	Neonatology	NA	Level 3	Fair
Muthamia[117]	2016	Kenya	Broader intervention/initiative	Independent	Unspecified health workers	Role-play, Synthetic PTT	Family planning	Partially	Level 3	Fair
Nataraja[118]	2022	Myanmar	Educational program	Independent	Doctors	Scenario-based, Mannequin	Surgery	Yes	Level 1; Level 2; Level 3	Good
Nelissen[119]	2017	Tanzania	Educational program	Standardized	Unspecified health workers, Nurse/midwives, Other	Scenario-based, Synthetic PTT	Obstetrics	Partially	Level 1; Level 2; Level 3	Good
Nevrekar[120]	2017	India	Educational program	Independent	Doctors	Unspecified simulation modality	Acute/critical care	No	Level 3	Fair
Nia[121]	2008	Iran	Broader intervention/initiative	Independent	Unspecified health workers, Other	Mannequin, Animal PTT	Acute/critical care	Yes	Level 3	Low
O'Hare[122]	2006	Uganda	Broader intervention/initiative	Independent	Nurses	Mannequin	Neonatology	Partially	None	Fair
Olson[123]	2012	Indonesia	Educational program	Standardized	Doctors, Nurses, Midwives	Mannequin, Synthetic PTT	Neonatology	No	Level 2; Level 3	Moderate
Opiyo[124]	2008	Kenya	Educational program	Standardized	Nurses, Midwives	Mannequin	Neonatology	NA	Level 3	Some concerns
Paludo[60]	2021	Brazil	Educational program	Independent	Doctors	Laparoscopic bench trainer, Extended reality	Surgery (Urology)	Partially	Level 3	Fair

Supplement to: Robinson SJA, Ritchie AMA, Pacilli M, Nestel D, McLeod E, Nataraja RM. Simulation-based education of health workers in low- and middle-income countries: a systematic review. *Glob Health Sci Pract*. 2024;12(6):e2400187. https://doi.org/10.9745/GHSP-D-24-000187

Author	Year	Country/Setting	Use of simulation-based education	Program Type	Learning Population/s	Simulation Modalities	Educational Focus	Statistically Significant Improvement in Results	Kirkpatrick Levels Evaluated (in Addition to Level 4)	Risk of bias / Quality Assessment Outcome
Petroze[125]	2015	Rwanda	Educational program	Standardized	Doctors, Nurses	Unspecified simulation modality	Acute/critical care; Communication/ leadership/ team training	Partially	Level 3	Poor
Pleah[126]	2016	1. Benin<br>2. Chad<br>3. Côte d'Ivoire<br>4. Niger<br>5. Senegal<br>6. Togo	Educational program	Independent	Unspecified health workers	Synthetic PTT	Family planning	NA	Level 3	Poor
Puchalski Ritchie[127]	2015	Malawi	Broader intervention/initiative	Independent	Nurses, Clinical Officers	Role-play	Infectious diseases	No	None	Low
Rule[43]	2017	Kenya	Broader intervention/initiative	Standardized	Unspecified health workers	Scenario-based, Mannequin	Neonatology	Partially	None	Good
Schmidt[128]	2018	Laos	Educational program	Independent	Unspecified health workers	Mannequin	Neonatology	Partially	None	Good
Schnittger[129]	2011	Malawi	Educational program	Independent	Doctors, Nurses, Midwives, Clinical Officers, Other	Mannequin, Synthetic PTT, Animal PTT	Acute/critical care; Obstetrics	NA	None	Fair
Shahnazi[130]	2021	Iran	Broader intervention/initiative	Independent	Unspecified health workers	Role-play	Communication/ leadership/ team training	Yes	Level 2; Level 3	Moderate
Shikuku[131]	2019	Kenya	Broader intervention/initiative	Independent	Midwives	Mannequin, Synthetic PTT	Obstetrics	Partially	Level 3	Moderate
Spitzer[132]	2014	Kenya	Educational program	Standardized	Unspecified health workers	Scenario-based, Mannequin	Neonatology; Obstetrics	Partially	Level 3	Fair
Størdal[47]	2020	Tanzania	Educational program	Standardized	Midwives	Scenario-based, Mannequin	Neonatology	Partially	Level 3	Fair
Sunguya[133]	2017	Tanzania	Educational program	Independent	Unspecified health workers	Role-play	Other: Nutrition	Yes	Level 2; Level 3	Some concerns
Tadesse[134]	2021	Ethiopia	Educational program	Standardized	Nurses, Midwives	Scenario-based	Neonatology	Partially	Level 1; Level 2; Level 3	Fair
Tilahun[135]	2017	Ethiopia	Broader intervention/initiative	Independent	Unspecified health workers	Synthetic PTT	Family planning	NA	Level 3	Fair
Tongprasert[136]	2010	Thailand	Educational program	Independent	Other	Other: Mixed PTT	Obstetrics	No	Level 3	Good

Supplement to: Robinson SJA, Ritchie AMA, Pacilli M, Nestel D, McLeod E, Nataraja RM. Simulation-based education of health workers in low- and middle-income countries: a systematic review. *Glob Health Sci Pract*. 2024;12(6):e2400187. https://doi.org/10.9745/GHSP-D-24-000187

Author	Year	Country/Setting	Use of simulation-based education	Program Type	Learning Population/s	Simulation Modalities	Educational Focus	Statistically Significant Improvement in Results	Kirkpatrick Levels Evaluated (in Addition to Level 4)	Risk of bias / Quality Assessment Outcome
Trevisanuto[137]	2015	Mozambique	Educational program	Standardized	Midwives	Scenario-based, Mannequin	Neonatology	No	Level 3	Good
Ullrich[138]	2020	Uganda	Educational program	Independent	Doctors	Cadaveric PTT	Surgery	No	Level 1; Level 2; Level 3	Good
Umunyana[42]	2020	Rwanda	Broader intervention/initiative	Standardized	Unspecified health workers	Scenario-based, Mannequin	Neonatology	Partially	Level 2; Level 3	Good
Van Den Broek[139]	2019	South Africa	Educational program	Standardized	Midwives, Unspecified health workers	Unspecified simulation modality	Neonatology; Obstetrics	No	Level 3	Some concerns
Van Heng[140]	2008	Cambodia	Educational program	Independent	Unspecified health workers	Animal PTT	Surgery	Yes	Level 3	Good
Varghese[141]	2016	India	Educational program	Independent	Doctors, Nurses	Role-play, Scenario-based, Mannequin	Neonatology; Obstetrics	No	Level 1; Level 2; Level 3	Moderate
Vossius[41]	2014	Tanzania	Educational program	Standardized	Unspecified health workers	Scenario-based, Mannequin	Neonatology	Yes	Level 1; Level 2; Level 3	Fair
Vukoja[62]	2021	1. Bosnia and Herzegovina<br>2. China<br>3. Dominican Republic<br>4. India<br>5. Ireland<br>6. Lebanon<br>7. Mexico<br>8. Pakistan<br>9. Philippines<br>10. Poland<br>11. Saudi Arabia<br>12. Serbia<br>13. Tanzania<br>14. Turkey<br>15. USA	Broader intervention/initiative	Independent	Doctors, Unspecified health workers	Telesimulation, Unspecified simulation modality	Acute/critical care	Yes	Level 3	Poor

Supplement to: Robinson SJA, Ritchie AMA, Pacilli M, Nestel D, McLeod E, Nataraja RM. Simulation-based education of health workers in low- and middle-income countries: a systematic review. *Glob Health Sci Pract*. 2024;12(6):e2400187. https://doi.org/10.9745/GHSP-D-24-000187

Author	Year	Country/Setting	Use of simulation-based education	Program Type	Learning Population/s	Simulation Modalities	Educational Focus	Statistically Significant Improvement in Results	Kirkpatrick Levels Evaluated (in Addition to Level 4)	Risk of bias / Quality Assessment Outcome
Walker[52]	2016	Mexico	Educational program	Standardized	Unspecified health workers	Scenario-based, Mannequin, Synthetic PTT, Hybrid simulation, In-situ simulation, Unspecified simulation modality	Neonatology; Obstetrics; Communication/ leadership/ team training	Partially	Level 3	Fair
Walker[53]	2020	1. Kenya<br>2. Uganda	Broader intervention/initiative	Standardized	Unspecified health workers	Scenario-based, Mannequin, Synthetic PTT, Hybrid simulation, In-situ simulation, Unspecified simulation modality	Neonatology; Obstetrics; Communication/ leadership/ team training	Yes	Level 3	Low
Walton[54]	2016	Guatemala	Broader intervention/initiative	Standardized	Unspecified health workers	Scenario-based, Mannequin, Synthetic PTT, Hybrid simulation, In-situ simulation, Unspecified simulation modality	Neonatology; Obstetrics	No	Level 3	Moderate
Wang[59]	2010	China	Educational program	Standardized	Doctors, Nurses	Scenario-based, Unspecified simulation modality	Acute/critical care	Partially	Level 3	Poor
Wendot[142]	2018	Kenya	Broader intervention/initiative	Independent	Doctors, Nurses, Clinical Officers	Role-play	Family planning	Partially	Level 3	Good

Supplement to: Robinson SJA, Ritchie AMA, Pacilli M, Nestel D, McLeod E, Nataraja RM. Simulation-based education of health workers in low- and middle-income countries: a systematic review. *Glob Health Sci Pract*. 2024;12(6):e2400187. https://doi.org/10.9745/GHSP-D-24-000187

Author	Year	Country/Setting	Use of simulation-based education	Program Type	Learning Population/s	Simulation Modalities	Educational Focus	Statistically Significant Improvement in Results	Kirkpatrick Levels Evaluated (in Addition to Level 4)	Risk of bias / Quality Assessment Outcome
Willcox[143]	2017	Ghana	Educational program	Independent	Midwives, Doctors, Nurses	Unspecified simulation modality	Neonatology; Obstetrics	Yes	None	Poor
Wrammert[45]	2017	Nepal	Educational program	Standardized	Doctors, Midwives	Scenario-based, Mannequin	Neonatology	Partially	Level 3	Good
Xu[144]	2012	China	Educational program	Standardized	Unspecified health workers	Mannequin	Neonatology	Yes	Level 2	Fair
Xu[145]	2014	China	Educational program	Standardized	Midwives, Doctors	Mannequin	Neonatology	Partially	Level 2; Level 3	Good
Yao[146]	2018	China	Educational program	Standardized	Doctors, Nurses	Scenario-based	Acute/critical care	No	Level 2; Level 3	Fair
Zaman[147]	2008	Pakistan	Educational program	Standardized	Other	Role-play, Scenario-based	Communication/ leadership/ team training	Partially	Level 3	Low

^PTT = Part-task trainer

References of included studies:

40. Goudar SS, Somannavar MS, Clark R, et al. Stillbirth and newborn mortality in India after helping babies breathe training. *Pediatrics*. 2013;131(2):e344-e352. CrossRef. Medline
41. Vossius C, Lotto E, Lyanga S, et al. Cost-effectiveness of the "helping babies breathe" program in a missionary hospital in rural Tanzania. *PLoS One*. 2014;9(7):e102080. CrossRef. Medline
42. Umunyana J, Sayinzoga F, Ricca J, et al. A practice improvement package at scale to improve management of birth asphyxia in Rwanda: a before-after mixed methods evaluation. *BMC Pregnancy Childbirth*. 2020;20(1):583. CrossRef. Medline
43. Rule ARL, Maina E, Cheruiyot D, et al. Using quality improvement to decrease birth asphyxia rates after 'Helping Babies Breathe' training in Kenya. *Acta Paediatr*. 2017;106(10):1666-1673. CrossRef. Medline
44. Kamath-Rayne BD, Josyula S, Rule ARL, Vasquez JC. Improvements in the delivery of resuscitation and newborn care after Helping Babies Breathe training. *J Perinatol*. 2017;37(10):1153-1160. CrossRef. Medline
45. Wrammert J, Kc A, Ewald U, Målqvist M. Improved postnatal care is needed to maintain gains in neonatal survival after the implementation of the Helping Babies Breathe initiative. *Acta Paediatr*. 2017;106(8):1280-1285. CrossRef. Medline
47. Størdal K, Eilevstjonn J, Mduma E, et al. Increased perinatal survival and improved ventilation skills over a five-year period: an observational study. *PLoS One*. 2020;15(10):e0240520. CrossRef. Medline

48. Bellad RM, Bang A, Carlo WA, et al. A pre-post study of a multi-country scale up of resuscitation training of facility birth attendants: does Helping Babies Breathe training save lives? *BMC Pregnancy Childbirth*. 2016;16(1):222. CrossRef. Medline
51. Afulani PA, Aborigo RA, Walker D, et al. Can an integrated obstetric emergency simulation training improve respectful maternity care? Results from a pilot study in Ghana. *Birth*. 2019;46(3):523-532. CrossRef. Medline
52. Walker DM, Cohen SR, Fritz J, et al. Impact evaluation of PRONTO Mexico: a simulation-based program in obstetric and neonatal emergencies and team training. *Simul Healthc*. 2016;11(1):1-9. CrossRef. Medline
53. Walker D, Otieno P, Butrick E, et al. Effect of a quality improvement package for intrapartum and immediate newborn care on fresh stillbirth and neonatal mortality among preterm and low-birthweight babies in Kenya and Uganda: a cluster-randomised facility-based trial. *Lancet Glob Health*. 2020;8(8):e1061-e1070. CrossRef. Medline
54. Walton A, Kestler E, Dettinger JC, et al. Impact of a low-technology simulation-based obstetric and newborn care training scheme on non-emergency delivery practices in Guatemala. *Int J Gynaecol Obstet*. 2016;132(3):359-364. CrossRef. Medline
55. Joudeh A, Ghosh R, Spindler H, et al. Increases in diagnosis and management of obstetric and neonatal complications in district hospitals during a high intensity nurse-mentoring program in Bihar, India. *PLoS One*. 2021;16(3):e0247260. CrossRef. Medline
56. Egenberg S, Masenga G, Bru LE, et al. Impact of multi-professional, scenario-based training on postpartum hemorrhage in Tanzania: a quasi-experimental, pre- vs. post-intervention study. *BMC Pregnancy Childbirth*. 2017;17(1):287. CrossRef. Medline
57. Crofts JF, Mukuli T, Murove BT, et al. Onsite training of doctors, midwives and nurses in obstetric emergencies, Zimbabwe. *Bull World Health Organ*. 2015;93(5):347-51. CrossRef. Medline
58. Dresang LT, Gonzalez MMA, Beasley J, et al. The impact of Advanced Life Support in Obstetrics (ALSO) training in low-resource countries. *Int J Gynaecol Obstet*. 2015;131(2):209-215. CrossRef. Medline
59. Wang P, Li NP, Gu YF, et al. Comparison of severe trauma care effect before and after advanced trauma life support training. *Chin J Traumatol*. 2010;13(6):341-4. Medline
60. Paludo AO, Knijnik P, Brum P, et al. Urology residents simulation training improves clinical outcomes in laparoscopic partial nephrectomy. *J Surg Educ*. 2021;78(5):1725-1734. CrossRef. Medline
61. Dean WH, Gichuhi S, Buchan JC, et al. Intense simulation-based surgical education for manual small-incision cataract surgery: the ophthalmic learning and improvement initiative in cataract surgery randomized clinical trial in Kenya, Tanzania, Uganda, and Zimbabwe. *JAMA Ophthalmol*. 2021;139(1):9-15. CrossRef. Medline
62. Vukoja M, Dong Y, Adhikari NKJ, et al. Checklist for early recognition and treatment of acute illness and injury: an exploratory multicenter international quality-improvement study in the ICUs with variable resources. *Crit Care Med*. 2021;49(6):e598-e612. CrossRef. Medline
63. Chan BT, Rauscher C, Issina AM, et al. A programme to improve quality of care for patients with chronic diseases, Kazakhstan. *Bull World Health Organ*. 2020;98(3):161-169. CrossRef. Medline

64. Hands S, Verriotis M, Mustapha A, et al. Nurse-led implementation of ETAT+ is associated with reduced mortality in a children's hospital in Freetown, Sierra Leone. *Paediatr Int Child Health*. 2020;40(3):186-193. CrossRef. Medline
65. Molyneux E, Ahmad S, Robertson A. Improved triage and emergency care for children reduces inpatient mortality in a resource-constrained setting. *Bull World Health Organ*. 2006;84(4):314-9. CrossRef. Medline
66. Irimu GW, Gathara D, Zurovac D, et al. Performance of health workers in the management of seriously sick children at a Kenyan tertiary hospital: before and after a training intervention. *PLoS One*. 2012;7(7):e39964. CrossRef. Medline
67. Gill CJ, Phiri-Mazala G, Guerina NG, et al. Effect of training traditional birth attendants on neonatal mortality (Lufwanyama Neonatal Survival Project): randomised controlled study. *BMJ*. 2011;342:d346. CrossRef. Medline
76. Arabi AME, Ibrahim SA, Manar AR, et al. Perinatal outcomes following Helping Babies Breathe training and regular peer-peer skills practice among village midwives in Sudan. *Arch Dis Child*. 2018;103(1):24-27. CrossRef. Medline
77. Arreola-Risa C, Mock C, Herrera-Escamilla AJ, et al. Cost-effectiveness and benefit of alternatives to improve training for prehospital trauma care in Mexico. *Prehosp Disaster Med*. 2004;19(4):318-325. CrossRef. Medline
78. Aziz K, Ma X, Lockyer J, et al. An evaluation of Acute Care of at-Risk Newborns (ACoRN), a Canadian education program, in Chinese neonatal nurseries. *Paediatr Child Health*. 2020;25(6):351-357. CrossRef. Medline
79. Baumgartner JN, Headley J, Kirya J, et al. Impact evaluation of a maternal and neonatal health training intervention in private Ugandan facilities. *Health Policy Plan*. 2021;36(7):1103-1115. CrossRef. Medline
80. Bender GJ, Kennally K. Implementing a neonatal transport system with simulation in Kosovo. *Air Med J*. 2016;35(3):126-131. CrossRef. Medline
81. Brathwaite KP, Bryce F, Moyer LB, et al. Evaluation of two newborn resuscitation training strategies in regional hospitals in Ghana. *Resusc Plus*. 2020;1-2:100001. CrossRef. Medline
82. Carlo WA, Goudar SS, Jehan I, et al. Newborn-care training and perinatal mortality in developing countries. *N Engl J Med*. 2010;362(7):614-23. CrossRef. Medline
83. Carvalho VS, Jr., Picanco MR, Volschan A, et al. Impact of simulation training on a telestroke network. *Int J Stroke*. 2019;14(5):500-507. CrossRef. Medline
84. Chang OH, Levy B, Lytle H, et al. Implementation of the alliance for innovation on maternal health program to reduce maternal mortality in Malawi. *Obstet Gynecol*. 2019;133(3):507-514. CrossRef. Medline
85. Charafeddine L, Badran M, Nakad P, et al. Strategic assessment of implementation of neonatal resuscitation training at a national level. *Pediatr Int*. 2016;58(7):595-600. CrossRef. Medline
86. Charyeva Z, Oguntunde O, Orobaton N, et al. Task shifting provision of contraceptive implants to community health extension workers: results of operations research in Northern Nigeria. *Glob Health Sci Pract*. 2015;3(3):382-394. CrossRef. Medline

87. Cioe-Pena EC, Granados JC, Wrightsmith LL, et al. Development and implementation of a hospital-based trauma response system in an urban hospital in San Salvador, El Salvador. *Trauma*. 2017;19(2):118-126. CrossRef
88. Clark M, Spry E, Daoh K, et al. Reductions in inpatient mortality following interventions to improve emergency hospital care in Freetown, Sierra Leone. *PLoS One*. 2012;7(9):e41458. CrossRef. Medline
89. Cummings MJ, Goldberg E, Mwaka S, et al. A complex intervention to improve implementation of World Health Organization guidelines for diagnosis of severe illness in low-income settings: a quasi-experimental study from Uganda. *Implement Sci*. 2017;12(1):126. CrossRef. Medline
90. Das R, Farzana FD, Sultana Z, et al. Evaluation of SIMESON, a training program to improve access to quality health care for pregnant women and newborn in different healthcare facilities of northern Bangladesh. *Nurs Open*. 2022;9(1):801-815. CrossRef. Medline
91. Das MK, Chaudhary C, Kaushal SK, et al. Impact of neonatal resuscitation capacity building of birth attendants on stillbirth rate at public health facilities in Uttar Pradesh, India. *Indian Pediatr*. 2019;56(5):369-373. Medline
92. de Melo BCP, Van der Vleuten CPM, Muijtjens AMM, et al. Effects of an in situ instructional design based postpartum hemorrhage simulation training on patient outcomes: an uncontrolled before-and-after study. *J Matern Fetal Neonatal Med*. 2021;34(2):245-252. CrossRef. Medline
93. Dumont A, Fournier P, Abrahamowicz M, et al. Quality of care, risk management, and technology in obstetrics to reduce hospital-based maternal mortality in Senegal and Mali (QUARITE): a cluster-randomised trial. *Lancet*. 2013;382(9887):146-57. CrossRef. Medline
94. Ellard DR, Shemdoe A, Mazuguni F, et al. Can training non-physician clinicians/associate clinicians (NPCs/ACs) in emergency obstetric, neonatal care and clinical leadership make a difference to practice and help towards reductions in maternal and neonatal mortality in rural Tanzania? The ETATMBA project. *BMJ Open*. 2016;6(2):e008999. CrossRef. Medline
95. Evans CL, Bazant E, Atukunda I, et al. Peer-assisted learning after onsite, low-dose, high-frequency training and practice on simulators to prevent and treat postpartum hemorrhage and neonatal asphyxia: a pragmatic trial in 12 districts in Uganda. *PLoS One*. 2018;13(12):e0207909. CrossRef. Medline
96. Fikree FF, Zerihun H. Scaling up a strengthened youth-friendly service delivery model to include long-acting reversible contraceptives in Ethiopia: a mixed methods retrospective assessment. *Int J Health Policy Manag*. 2020;9(2):53-64. CrossRef. Medline
97. Gandhi Bhatt A, Dole KS, Deshpande M, et al. Impact of a structured training program to enhance skills in phacoemulsification surgery. *Indian J Ophthalmol*. 2021;69(12):3697-3702. CrossRef. Medline
98. Geldenhuys L, Wise R, Rodseth R. The impact of a bundled intrahospital transfer protocol on the safety of critically ill patients in a South African metropolitan hospital system. *Southern Afr J Anaesthesia Analgesia*. 2020;26(3):139-148. CrossRef
99. Goudar SS, Dhaded SM, McClure EM, et al. ENC training reduces perinatal mortality in Karnataka, India. *J Matern Fetal Neonatal Med*. 2012;25(6):568-74. CrossRef. Medline
100. Gueye B, Wesson J, Koumtingue D, et al. Mentoring, Task sharing, and community outreach through the TutoratPlus approach: increasing use of long-acting reversible contraceptives in Senegal. *Glob Health Sci Pract*. 2016;4(Suppl 2):S33-S43. CrossRef. Medline

101. Hashmi ZG, Haider AH, Zafar SN, et al. Hospital-based trauma quality improvement initiatives: first step toward improving trauma outcomes in the developing world. *J Trauma Acute Care Surg*. 2013;75(1):60-68. CrossRef. Medline
102. Hawkins L, Gertz AM, Badubi O, et al. Integration of family planning services into health care for HIV-positive women in Botswana. *Int J Gynaecol Obstet*. 2021;152(2):208-214. CrossRef. Medline
103. Houy C, Ha SO, Steinholt M, et al. Delivery as trauma: a prospective time-cohort study of maternal and perinatal mortality in rural Cambodia. *Prehosp Disaster Med*. 2017;32(2):180-186. CrossRef. Medline
104. Hu LQ, Flood P, Li Y, et al. No pain labor & delivery: a global health initiative's impact on clinical outcomes in China. *Anesth Analg*. 2016;122(6):1931-8. CrossRef. Medline
105. Innerdal M, Simaga I, Diall H, et al. Reduction in perinatal mortality after implementation of HBB training at a district hospital in Mali. *J Trop Pediatr*. 2020;66(3):315-321. CrossRef. Medline
106. Jones-Konneh TEC, Murakami A, Sasaki H, et al. Intensive education of health care workers improves the outcome of Ebola virus disease: lessons learned from the 2014 outbreak in Sierra Leone. *Tohoku J Exp Med*. 2017;243(2):101-105. CrossRef. Medline
107. Jorro-Baron F, Suarez-Anzorena I, Burgos-Pratx R, et al. Handoff improvement and adverse event reduction program implementation in paediatric intensive care units in Argentina: a stepped-wedge trial. *BMJ Quality & Safety*. 2021;30(10):782-791. CrossRef. Medline
108. Kestler E, Ambrosio G, Hemming K, et al. An integrated approach to improve maternal and perinatal outcomes in rural Guatemala: a stepped-wedge cluster randomized trial. *Int J Gynaecol Obstet*. 2020;151(1):109-116. CrossRef. Medline
109. Matendo R, Engmann C, Ditekemena J, et al. Reduced perinatal mortality following enhanced training of birth attendants in the Democratic Republic of Congo: a time-dependent effect. *BMC Med*. 2011;9:93. CrossRef. Medline
110. Michelangelo H, Angriman F, Pizarro R, et al. Implementation of an experiential learning strategy to reduce the risk of ventilator-associated pneumonia in critically ill adult patients. *J Intensive Care Soc*. 2020;21(4):320-326. CrossRef. Medline
111. Miller PC, Rashida G, Tasneem Z, et al. The effect of traditional birth attendant training on maternal and neonatal care. *Int J Gynaecol Obstet*. 2012;117(2):148-52. CrossRef. Medline
112. Mogilevkina I, Gurianov V, Lindmark G. Effectiveness of emergency obstetric care training at the regional level in Ukraine: a non-randomized controlled trial. *BMC Pregnancy Childbirth*. 2022;22(1):145. CrossRef. Medline
113. Mould-Millman NK, Dixon J, Lamp A, et al. A single-site pilot implementation of a novel trauma training program for prehospital providers in a resource-limited setting. *Pilot Feasibility Stud*. 2019;5(1):143. CrossRef. Medline
114. Mubeen K, Baig M, Abbas S, et al. Helping babies breathe: assessing the effectiveness of simulation-based high-frequency recurring training in a community-based setting of Pakistan. *BMC Pediatr*. 2021;21(1):555. CrossRef. Medline
115. Mugore S, Kassouta NT, Sebikali B, et al. Improving the quality of postabortion care services in Togo increased uptake of contraception. *Glob Health Sci Pract*. 2016;4(3):495-505. CrossRef. Medline

116. Mukhtar-Yola M, Audu LI, Olaniyan O, et al. Decreasing birth asphyxia: utility of statistical process control in a low-resource setting. *BMJ Open Quality*. 2018;7 (3) (e000231):(no pagination). CrossRef. Medline
117. Muthamia M, Owino K, Nyachae P, et al. The Tupange project in Kenya: a multifaceted approach to increasing use of long-acting reversible contraceptives. *Glob Health Sci Pract*. 2016;4(Suppl 2):S44-S59. CrossRef. Medline
118. Nataraja RM, Yin Mar O, Ljuhar D, et al. Long-term impact of a low-cost paediatric intussusception air enema reduction simulation-based education programme in a low-middle income country. *World J Surg*. 2022;46(2):310-321. CrossRef. Medline
119. Nelissen E, Ersdal H, Mduma E, et al. Clinical performance and patient outcome after simulation-based training in prevention and management of postpartum haemorrhage: an educational intervention study in a low-resource setting. *BMC Pregnancy Childbirth*. 2017;17(1):301. CrossRef. Medline
120. Nevrekar V, Panda PK, Wig N, et al. An interventional quality improvement study to assess the compliance to cardiopulmonary resuscitation documentation in an Indian teaching hospital. *Indian J Crit Care Med*. 2017;21(11):758-764. CrossRef. Medline
121. Nia MS, Naffisi N, Mohebbi HA, et al. The role of performing life support courses in rural areas in improving pre-hospital physiologic conditions of patients with penetrating injuries. *J Coll Physicians Surg Pak*. 2008;18(9):538-541. Medline
122. O'Hare BA, Nakakeeto M, Southall DP. A pilot study to determine if nurses trained in basic neonatal resuscitation would impact the outcome of neonates delivered in Kampala, Uganda. *J Trop Pediatr*. 2006;52(5):376-9. CrossRef. Medline
123. Olson KR, Caldwell A, Sihombing M, et al. Community-based newborn resuscitation among frontline providers in a low-resource country. *Int J Gynaecol Obstet*. 2012;119(3):244-7. CrossRef. Medline
124. Opiyo N, Were F, Govedi F, et al. Effect of newborn resuscitation training on health worker practices in Pumwani Hospital, Kenya. *PLoS One*. 2008;3(2):e1599. CrossRef. Medline
125. Petroze RT, Byiringiro JC, Ntakiyiruta G, et al. Can focused trauma education initiatives reduce mortality or improve resource utilization in a low-resource setting? *World J Surg*. 2015;39(4):926-33. CrossRef. Medline
126. Pleah T, Hyjazi Y, Austin S, et al. Increasing use of postpartum family planning and the postpartum IUD: early experiences in West and Central Africa. *Glob Health Sci Pract*. 2016;4(Suppl 2):S140-S152. CrossRef. Medline
127. Puchalski Ritchie LM, Schull MJ, Martiniuk AL, et al. A knowledge translation intervention to improve tuberculosis care and outcomes in Malawi: a pragmatic cluster randomized controlled trial. *Implement Sci*. 2015;10:38. CrossRef. Medline
128. Schmidt S, Bounnack S, Hoehn T. Neonatal mortality and morbidity in the post-implementation period of a neonatal teaching program in provincial hospitals in Laos. *Public Health*. 2018;154:123-129. CrossRef. Medline
129. Schnittger T, Downie P, Pollach G. The effect of providing resuscitation training to frontline staff on rates of maternal and trauma mortality in two health districts in Malawi. *Malawi Med J*. 2011;23(1):11-5. CrossRef. Medline
130. Shahnazi H, Araban M, Karimy M, et al. A quasi-experimental study to improve health service quality: implementing communication and self-efficacy skills training to primary healthcare workers in two counties in Iran. *BMC Med Educ*. 2021;21(1):369. CrossRef. Medline

131. Shikuku DN, Mukosa R, Peru T, et al. Reducing intrapartum fetal deaths through low-dose high frequency clinical mentorship in a rural hospital in Western Kenya: a quasi-experimental study. *BMC Pregnancy Childbirth*. 2019;19(1):518. CrossRef. Medline
132. Spitzer RF, Steele SJ, Caloia D, et al. One-year evaluation of the impact of an emergency obstetric and neonatal care training program in Western Kenya. *Int J Gynaecol Obstet*. 2014;127(2):189-93. CrossRef. Medline
133. Sunguya BF, Mlunde LB, Urassa DP, et al. Improving feeding and growth of HIV-positive children through nutrition training of frontline health workers in Tanga, Tanzania. *BMC Pediatr*. 2017;17(1):94. CrossRef. Medline
134. Tadesse M, Hally S, Rent S, et al. Effect of a low-dose/high-frequency training in introducing a nurse-led neonatal advanced life support service in a referral hospital in Ethiopia. *Front Pediatr.* 2021;9:777978. CrossRef. Medline
135. Tilahun Y, Lew C, Belayihun B, et al. Improving contraceptive access, use, and method mix by task sharing implanon insertion to frontline health workers: the experience of the integrated family health program in Ethiopia. *Glob Health Sci Pract*. 2017;5(4):592-602. CrossRef. Medline
136. Tongprasert F, Wanapirak C, Sirichotiyakul S, et al. Training in cordocentesis: the first 50 case experience with and without a cordocentesis training model. *Prenatal Diagnosis*. 2010;30(5):467-70. CrossRef. Medline
137. Trevisanuto D, Bertuola F, Lanzoni P, et al. Effect of a neonatal resuscitation course on healthcare providers' performances assessed by video recording in a low-resource setting. *PLoS One*. 2015;10(12):e0144443. CrossRef. Medline
138. Ullrich SJ, DeWane MP, Cheung M, et al. Development of an operative trauma course in Uganda-a report of a three-year experience. *J Surg Res*. 2020;256:520-527. CrossRef. Medline
139. Van Den Broek N, Ameh C, Madaj B, et al. Effects of emergency obstetric care training on maternal and perinatal outcomes: a stepped wedge cluster randomised trial in South Africa. *BMJ Global Health*. 2019;4(6):e001670. CrossRef. Medline
140. Van Heng Y, Davoung C, Husum H. Non-doctors as trauma surgeons? A controlled study of trauma training for non-graduate surgeons in rural Cambodia. *Prehosp Disaster Med*. 2008;23(6):483-489. CrossRef. Medline
141. Varghese B, Krishnamurthy J, Correia B, et al. Limited effectiveness of a skills and drills intervention to improve emergency obstetric and newborn care in Karnataka, India: a proof-of-concept study. *Glob Health Sci Pract*. 2016;4(4):582-593. CrossRef. Medline
142. Wendot S, Scott RH, Nafula I, et al. Evaluating the impact of a quality management intervention on post-abortion contraceptive uptake in private sector clinics in Western Kenya: a pre- and post-intervention study. *Reprod Health*. 2018;15(1):10. CrossRef. Medline
143. Willcox M, Harrison H, Asiedu A, et al. Incremental cost and cost-effectiveness of low-dose, high-frequency training in basic emergency obstetric and newborn care as compared to status quo: part of a cluster-randomized training intervention evaluation in Ghana. *Global Health*. 2017;13(1):88. CrossRef. Medline
144. Xu T, Wang HS, Ye HM, et al. Impact of a nationwide training program for neonatal resuscitation in China. *Chin Med J*. 2012;125(8):1448-1456. Medline
145. Xu T, Wang H, Gong L, et al. The impact of an intervention package promoting effective neonatal resuscitation training in rural China. *Resuscitation*. 2014;85(2):253-259. CrossRef. Medline

146. Yao JL, Zhou CX, Fu JM, et al. A survey on the epidemiology of trauma and China trauma care training in subtropical regions of Hainan Province. *J Acute Dis*. 2018;7(3):108-114. CrossRef
147. Zaman S, Ashraf RN, Martines J. Training in complementary feeding counselling of healthcare workers and its influence on maternal behaviours and child growth: a cluster-randomized controlled trial in Lahore, Pakistan. *J Health Pop Nutr*. 2008;26(2):210-222. Medline
